# Detection of Methicillin-resistant *Staphylococcus aureus* (MRSA) and biofilm formation among dental patients and dental health care workers: cross sectional study

**DOI:** 10.1007/s00784-025-06684-9

**Published:** 2026-01-03

**Authors:** Manal M. Darwish, Nashwa Naguib Omar, Rania Farouk, Kawther Ibrahim, Rasha Attia, Lamiaa M. EL-Moussely

**Affiliations:** 1 Microbiology and Immunology Department, Faculty of Pharmacy, October University for Modern Sciences and Arts, Giza, Egypt; 2 Oral Medicine and Periodontology Department, Faculty of Dentistry, October University for Modern Sciences and Arts, Giza, Egypt; 3 Clinical Pathology Department, Faculty of Medicine, Ain Shams University, Cairo, Egypt; 4 Medical Microbiology and Immunology Department, Faculty of Medicine, Ain Shams University, Cairo, Egypt; 5 Microbiology and Immunology Department, Faculty of Medicine, Benha National University, Abour City, Egypt

**Keywords:** MRSA, Biofilms, Dental staff, Congo red, *S. aureus*, Microtiter plate

## Abstract

**Objectives:**

Dental clinics can harbor and transmit Methicillin-resistant *Staphylococcus aureus* (MRSA) which is a challenging antibiotic-resistant pathogen with global health implications. The aim of the study was to assess the prevalence, resistance patterns and biofilm formation by *Staphylococcus aureus (S. aureus)* isolated from dental patients and dental health care workers (DHCWs) .

**Materials and methods:**

Two hundred nasal swab specimens were collected aseptically from 120 dental patients and 80 DHCWs across Egypt including Cairo, Giza, and Upper Egypt over a 12 –month period. The study incorporated a comprehensive analysis to identify MRSA isolates using disk diffusion test with cefoxitin, to confirm MRSA isolates by molecular detection of the *mecA* gene and the prevalence intercellular adhesion gene A and D (*icaA* and *icaD*) using Polymerase chain reaction assays, to evaluate the antimicrobial resistance profiles of *S. aureus* using Kirby-Bauer disk diffusion method, and to detect the qualitative and quantitative characteristics of the biofilm formation using Congo red agar plates and microtiter plate method respectively. Statistical package for social science was used for data analysis.

**Results:**

Results were interpreted following the Clinical and Laboratory Standards Institute 2023 guidelines. *S. aureus* was identified in 47.1% of the collected *Staphylococcus* species with 66.6% of these *S. aureus* isolates carried *mecA* genes and identified as MRSA. The identification rate of *icaA* and *icaD* in MRSA isolates was significant compared to Methicillin-Sensitive *Staphylococcus aureus* (MSSA) isolates, with detection rates of 88.6% and 40.9% respectively. Regarding antimicrobial resistance patterns, all isolates were resistant to Penicillin (100%) with notable resistance to other antibiotics including Clindamycin (60.6%), Erythromycin (42.4%), and Gentamicin (24.2%). Biofilm formation ability was detected in 72.7% of the MRSA isolates with high incidence of strong and moderate biofilm formation in 93.1% of these isolates compared to MSSA isolates (50%).

**Conclusions:**

The high MRSA colonization rates among DHCWs and patients, along with significant antibiotic resistance and biofilm-forming abilities, highlight the urgent need for regular screening and enhanced infection control measures in dental healthcare settings. Future research should aim to expand sampling and clarify more site selection criteria to better inform infection control strategies and improve generalizability.

**Clinical relevance:**

Given the persistent threat of MRSA in dental settings, the implementation of rigorous infection control protocols and comprehensive antibiotic stewardship strategies is imperative to mitigate transmission risks and enhance patient safety.

**Supplementary Information:**

The online version contains supplementary material available at 10.1007/s00784-025-06684-9.

## Introduction

*S. aureus* colonizes various body parts as a commensal flora including anterior nares, skin, and oral cavity. However nasal epithelium is the predominant colonization area, where carriers reach an average of 20–40% in adults [1]. Globally, with the wide spread of empirical antimicrobial prescriptions and antimicrobials misuse and over usage especially in low- and middle-income countries [2], *S. aureus* acquired resistance to a wide range of antimicrobials, mostly to β-lactams in addition to tetracyclines, fluoroquinolones, macrolides, aminoglycosides and lincosamides [3].

MRSA is a global health threat, leading to significant morbidity and mortality rates [4]. MRSA accounts for up to 90% of *S. aureus* infections, rendering standard antibiotic treatments ineffective. According to the World Health Organization (WHO), MRSA has been detected in every province across the globe, and infections caused by this resistant strain are associated with a 64% higher risk of mortality compared to infections caused by drug-sensitive strains, largely due to its resistance to commonly used antibiotics [5]. Its longstanding status as a confirmed pathogen in severe nosocomial infections with its resistance to antimicrobials, complicates treatment, raises mortality rates, and causes prolonged hospital stays and increased healthcare costs [6]. MRSA infections are classified into healthcare-associated (HA-MRSA) and community-associated (CA-MRSA). HA-MRSA typically occurs in patients with recent hospitalization, surgery, or medical device use, and is often multidrug-resistant. In contrast, CA-MRSA affects healthy individuals outside of healthcare settings, usually causing skin and soft tissue infections. It spreads through direct contact and is often less resistant but more virulent [7].

MRSA disseminates by direct contact with droplets, open wounds, colonized skin and shared equipment or surfaces [8]. Therefore, healthcare personnel in contact with MRSA carriers have high risk for MRSA acquisition [9]. In dental clinics, MRSA is primarily transmitted through direct contact with colonized or infected patients most commonly via saliva, nasal secretions, or skin lesions and indirectly through contaminated instruments, gloves, or surfaces. While transmission through blood is uncommon, the risk of MRSA transmission via contaminated surgical instruments exists, particularly if those instruments encounter blood and are not properly sterilized reinforcing the importance of strict infection control and sterilization protocols [10].

Methicillin resistance is mediated by a mutated protein called penicillin-binding protein (PBP2a) encoded by *mecA* gene and is found in a chromosomal mobile genetic element called staphylococcal cassette chromosome *mec* (*SCCmec*) [11]. This genetic element also encodes the regulatory genes, *mecR1* and *mecI*. After being exposed to β-lactam antibiotics, *mecR1* cleaves *mecI*, disrupting its binding to the *mecA* promoter allowing production of PBP2a. Consequently, cell wall synthesis is carried on despite the presence of inhibitory concentrations of β-lactams, preventing cell death and lysis [12].

*S. aureus* biofilm formation is a significant virulence factor, and it is associated with persistent chronic infections and high morbidity and mortality rates [13, 14]. Biofilms significantly enhance *S. aureus* resistance to antimicrobials not only by creating a protective environment that limits drug penetration but also by facilitating the horizontal transfer of antibiotic resistance genes, such as insertion sequences, within the protective biofilm matrix. This environment promotes gene exchange among bacterial cells, contributing to increased antimicrobial resistance [15]. The adhesion-promoting polysaccharide intercellular adhesin (*PIA*) protein, encoded by the *icaADBC* gene locus (specifically, the *icaA* and *icaD* genes), plays an important role in mediating adhesion and augmenting biofilm formation especially in cases with indwelling medical devices [16].

Dental clinics as a part of healthcare settings can serve as potential reservoir for MRSA and DHCWs may serve as MRSA carriers, with potential transmission to patients or other DHCWs [17]. Staff awareness and strict infection control measures can reduce MRSA in dental settings and early detection of MRSA in dental clinics is crucial for timely intervention, encompassing appropriate treatment and robust infection control measures [18].

In comparison to studies on MRSA isolates from hospitals, there is limited number of studies focusing on MRSA in dental settings regarding the prevalence, the biofilm formation ability, and antimicrobial resistance profiles of MRSA isolated from these settings. Studies on MRSA isolates from dental settings could provide critical insights into infection control practices and in appropriate antibiotics selection.

With a focus on MRSA isolates from dental settings in Egypt, this current study provided a complete analysis by including the antimicrobial resistance profile of *S. aureus*, conducting phenotypic biofilm formation assays, and employing Polymerase chain reaction (PCR) techniques to detect key genetic markers—*mecA* for antimicrobial resistance, and *icaA* and *icaD* for biofilm formation. It also reports the prevalence of MRSA in dental clinics across both Upper and Lower Egypt, serving as a foundational step for future research and antibiotic stewardship efforts in dental settings.

## Materials and methods

### Study design

This cross-sectional study was conducted between March 2023 and February 2024 and the investigations were carried out in the microbiology laboratories of October University for Modern Sciences and Arts (MSA) and Ain Shams University-Egypt. Ethical approval was obtained from the Ethics Committee of Faculty of Pharmacy at MSA University (Approval number: M1/Ec1/2023PD).

### Sample size calculation

Epi-Calc 2000 was used to determine the sample size for this study based on data reported from a previous study on the prevalence of MRSA isolates in different dental care settings in Egypt by Khairalla et al. [19], which included 88 specimens from the anterior nares of both patients and DHCWs. Assuming 80% power, 0.05 level of significance, 60.5% null hypothesis value and estimated proportion of 70.6%, sample size was 179 participants. Considering the drop-out rate of 10%, therefore the final sample size will be 197 participants.

### Study population

Participants were dental patients and DHCWs that were recruited from various dental clinics across Egypt. Participants excluded from the study were those who undergone nasal decolonization with mupirocin cream, received antibiotics within the previous 72 h. Informed written consent was obtained before their voluntarily participation. Their demographic data (including profession, age, sex), smoking habits, prior hospital admissions, chronic disease history, recent antibiotic use, and mupirocin application, was gathered through a structured questionnaire formulated for the study (Appendix 1).

### Microbiological investigations

#### Sample collection and transport

A total of 200 nasal swab specimens were collected aseptically, including 120 samples from dental patients and 80 from DHCWs. These specimens were promptly placed in nutrient broth for transport, ensuring viability then transported to Microbiology laboratories for further analysis [20].

#### Isolation, identification, and antibiotic susceptibility of *S. aureus*

After incubation of the samples, the initial suspension was streaked onto mannitol salt agar plates. After 24 h incubation at 35° C., putative yellow zone *S. aureus* colonies were picked and re-streaked onto Trypticase Soy Agar (TSA; Lab M, UK) to check purity as TSA ensures that a single colony represents a pure culture as mixed cultures may arise during sub culturing on mannitol salt agar [21]. Then identified biochemically by catalase and coagulase and microscopy to detect the *S. aureus* phenotype.

Antimicrobial susceptibility testing using Kirby-Bauer disk diffusion method was performed on all *S. aureus* isolates using various antimicrobial discs (Oxoid, UK.), including β-lactams (penicillin 1unit and cefoxitin 30ug), glycopeptides (vancomycin 30ug), aminoglycosides (gentamicin 10ug and amikacin 30ug), macrolides (erythromycin 15ug), tetracyclines (tetracycline 30ug), fluoroquinolones (ciprofloxacin 5ug and moxifloxacin 5ug), lincosamides (clindamycin 2ug), folate pathway inhibitors (trimethoprim-sulfamethoxazole 1.25/23.75ug), phenicol (chloramphenicol 30ug), and oxazolidinones (linezolid 10ug). Results were interpreted following the Clinical and Laboratory Standards Institute (CLSI) 2023 guidelines [22]. Although vancomycin disc diffusion is not recommended for determining susceptibility in MRSA isolates according to CLSI guidelines, it was introduced here as a preliminary screening step for general antibiotic sensitivity among staphylococcus species.

#### Screening of MRSA

MRSA isolates were identified through the disk diffusion method using cefoxitin (30 µg). Subsequently, PCR targeting the *mecA* gene was conducted to confirm the isolates. Molecular detection of *mecA* gene detection by PCR using Taq DNA Polymerase Kit (Thermo Fisher Scientific, USA) was performed as described by Merlino et al., to detect the presence of *mecA* gene, corresponding to a 533 bp fragment [23]. The protocol was followed with slight modifications, including an initial denaturation at 95 °C for 5 min, followed by 35 cycles of amplification at 95 °C for 30 s, 52 °C for 60 s, 72 °C for 60 s, and storage at 4 °C.

#### Phenotypic characterization of biofilm production

##### Qualitative measurement by congo red agar assay (CRA)

To determine biofilm formation, isolates were cultured on CRA plates then they were aerobically incubated at 37 °C for 24 h. Biofilm-producing strains appeared as black colonies, while non-biofilm producers formed red colonies [24].

##### Quantitative measurement by microtiter plate method (MTP)

After incubating bacterial cultures in a 96-well polystyrene microplate at 37 °C for 24 h, microbial growth and turbidity were assessed by measuring the optical density (OD) at 600 nm using an ELISA reader. This step provides an initial OD value reflecting total bacterial growth, including both planktonic and sessile cells, which is essential for normalizing subsequent biofilm quantification data.

Following this step, planktonic cells were removed and washing the wells three times with phosphate-buffered saline (PBS) were done to eliminate non-adherent cells. The remaining adherent biofilm was fixed by adding 200 µL of 99% ethanol to each well for 15 min at room temperature then ethanol was removed, and the wells were allowed to air-dry completely. Once dried, 200 µL of 0.1% crystal violet solution was added to each well for 10 min at room temperature to stain the biofilms. After staining, the wells were gently rinsed with distilled water multiple times to remove unbound dye, then the residual liquid was eliminated by inverting the plate on absorbent paper. To quantify the biofilm, 200 µL of 30% acetic acid was added to each well for 10–15 min at room temperature with gentle agitation to solubilize the bound crystal violet dye, then 125 µL of the solubilized dye was transferred to a new microtiter plate for absorbance measurement at 590 nm using a microplate reader [25, 26].

The cut-off optical density (ODc) value was determined to differentiate biofilm-forming capabilities among *S. aureus* isolates. To establish the ODc, an uninoculated medium and a known biofilm non-producing strain were used as negative controls. Multiple replicates of the negative control were measured spectrophotometrically to obtain baseline OD readings. The ODc then was calculated as the mean OD of the negative control plus three times its standard deviation (ODc = mean OD + 3 × SD). Results were categorized into groups: (I) OD ≤ ODc indicated no biofilm production, (II) ODc < OD ≤ (2 × ODc) denoted weak biofilm production, (III) (2 × ODc) < OD ≤ (4 × ODc) represented moderate biofilm production, and (IV) (4 × ODc) < OD indicated strong biofilm production [27]. Quality control using reference strains *(S. aureus* strain ATCC 25923 and *S. epidermidis* strain ATCC 12228) was done [28].

##### Molecular detection of *IcaA* and *IcaD* genes responsible for PIA synthesis

*S. aureus* chromosomal DNA samples were extracted using the Thermo Scientific Genomic DNA Purification Kit (Catalog number K0512). The primers for amplifying the *icaA* and *icaD* genes were obtained from Invitrogen Fisher Scientific (United States) based on the published sequence of the ica locus as outlined by Cramton et al. [29]. The *icaA* gene primers were Forward: 5′-TCTCTTGCAGGAGCAATCAA-3′ and Reverse: 5′-TCAGGCACTAACATCCAGCA-3′, producing a 450 bp amplicon. The *icaD* gene primers were Forward: 5′-ATGGTCAAGCCCAGACAGAG-3′ and Reverse: 5′-CGTGTTTTCAACATTTAATGCAA-3′, generating a 184 bp amplicon. Ten microliters of extracted DNA was used as a template in a 50 µL PCR reaction mixture, which contained 25 µL of Taq Master Mix (2X) (Thermo Scientific), 5 µL of a 10 µM stock solution of each primer, and nuclease-free water to bring the total volume to 50 µL. PCR amplification of the *icaA* and *icaD* genes was performed as follows: thirty cycles of amplification were carried out, with each cycle consisting of denaturation at 92 °C for 45 s, annealing at 49 °C for 45 s, and elongation at 72 °C for 45 s. A final extension step was carried out at 72 °C for 7 min. The amplification was performed in a thermocycler (Eppendorf, USA). *S. aureus* ATCC 25,923 was used as a positive control for both genes, and a negative control (no-template control, NTC) was included in each PCR run. Electrophoresis was performed in 1.5% (wt/vol) agarose gel stained with ethidium bromide.

### Statistical methods

Statistical package for social science (SPSS version 24) was used for data analysis. Normality was assessed using the Shapiro-Wilk test. Normally distributed quantitative data were described using arithmetic mean and standard deviation, while abnormally distributed data were described using median and interquartile range. Bivariate relationship was performed using the chi-square and Fisher’s exact tests where appropriate. T-independent was used to compare normally distributed quantitative data and Mann-Whitney for skewed data. P value was calculated to assess statistical significance, a value less than 0.05 was considered statistically significant.

## Results

### Demographic data of the participants

The clinical specimens included 200 nasal swabs collected from 120 dental patients and 80 DHCWs including 42 dentists and 38 dental assistants. Participants were aged between 18 and 60 years with average age of 35 years and were recruited from different dental clinics across Egypt including 131 specimens from Lower Egypt governorates (Cairo and Giza) and 69 specimens from Upper Egypt governorates (Assiut and Luxor).

### Prevalence and antibiotic susceptibility of MRSA

In this study, 140 (70%) isolates from the total 200 specimens collected were *Staphylococcus* species; **(140/200).** Among the total 140 *Staphylococcus* species isolates, 66 (47.1%) were *S. aureus* (16 isolates were from DHCWs and 50 isolates from dental patients); **(66/140)**, while 74 (52.9%) were coagulase-negative *Staphylococcus* (CoNS); **(72/140).** MRSA isolates were identified in 66.6% of the total *S. aureus* isolates; **(44/66)**, while MSSA isolates were 36.4% **(22/66)**.

MRSA prevalence among participants is presented in Table (1). The results of the antimicrobial sensitivity tests of MRSA and MSSA against different antimicrobial agents are represented in Table (2).

**Table 1:** Prevalence of the MRSA isolates in relation to the demographic data of the participants

Participants demographic data	Items	Number and percentage (%) of MRSA isolates
**Location**	Upper Egypt	15/44 (34.5%)
	Lower Egypt	29/44 (65.5%)
**Category**	DHCWs	5/44 (11.4%)
	Dental patients	39/44 (88.6%)
**Education level**	Low educated participants	25/44(56.8%)
	High educated participants	19/44(31.8%) (5/19 were DHCWs)
**Gender**	Male	29/44 (65.9%)
	Female	15/44 (34.1%)
**Smoking status**	Smokers	32/44 (72.7%)
	Non-smokers	12/44 (27.2%)
**Previous hospitalization**	Yes	28/44 (63.6%)
	No	16/44 (36.6%)

**Table 2:** Antimicrobial susceptibility testing of MRSA isolates versus MSSA isolates presented in number and percentage (%)

Antimicrobial group	Antimicrobial agent	Resistant MRSA isolates	Resistant MSSA isolates
β-lactams	Penicillin Cefoxitin	44 (100%) 44 (100%)	22(100%) 0%
Glycopeptides	Vancomycin	1 (2.3%)	1 (4.5%)
Aminoglycosides	Gentamicin Amikacin	13 (29%) 2 (4.5%)	3 (13.6%) 1 (4.5%)
Tetracyclines	Tetracyclines	6 (13.6%)	2 (9.1%)
Fluoroquinolones	Ciprofloxacin Moxifloxacin	11(25%) 8 (18.2%)	3 (13.6%) 4 (18.2%)
Macrolides	Erythromycin	23 (52.3%)	5 (22.7%)
Lincosamides	Clindamycin	32 (72.7%)	8 (36.4%)
Phenicols	Chloramphenicol	11(25%)	5 (22.7%)
Folate pathway inhibitors	Trimethoprim sulfamethoxazole	4 (9.1%)	2 (9.1%)
Oxazolidinones	Linezolid	3 (6.8%)	2 (9.1%)

### Biofilm production

#### CRA assay result

Among the *S. aureus* strains, 72.7% **(48/66)** were identified as biofilm producers, forming rough black colonies, while 27.3% **(18/66)** were non-biofilm producers, displaying smooth red colonies. As anticipated, the reference strains ATCC 25,923 and ATCC 12,228 demonstrated positive and negative biofilm production, respectively.

#### MTP result

93.1% **(41/44)** of MRSA isolates showed strong to moderate biofilm formation ability compared to 50% **(11/22)** of the MSSA isolates with 2.3% and 22.7% weak biofilm formation ability for MRSA and MSSA respectively as shown in Table (3).

**Table 3:** Biofilm production ability of MRSA and MSSA isolates presented in number and percentage (%)

Isolates	Strong biofilm producer	Moderate biofilm producer	Weak biofilm producer	Non-Biofilm formation	Total biofilm formation ability	*P* value
MRSA	16/44 (36.3%)	25/44 (56.8%)	1/44 (2.3%)	2/44 (4.5%)	42/44 (95.4%)	*P*= 0.0008**
MSSA	3/22 (13.6%)	8/22 (36.4%)	5/22 (22.7%)	6/22 (27.3%)	16/22 (72.2%)	

**p-value < 0.001 is highly significant

### Genetic markers detection

**Fig. 1:**
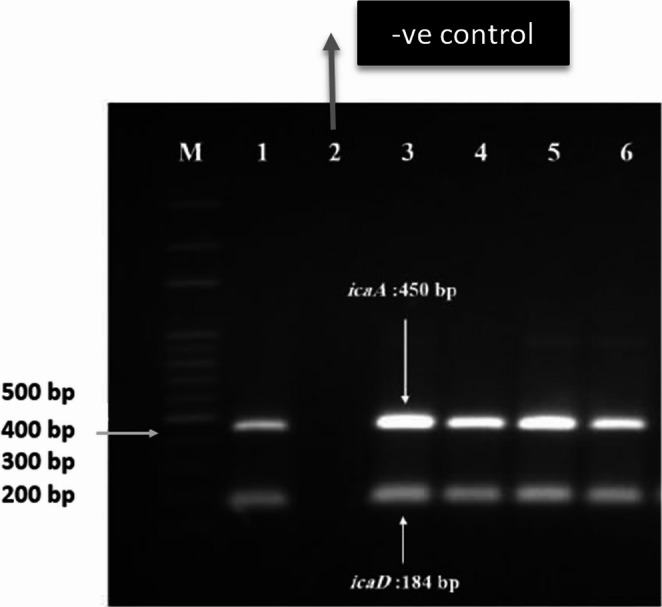
PCR detection of ***icaA*** and ***icaD*** genes; Lane 1: ***S. aureus*** positive control ATCC 25,923, Lane 2: Reaction control, and Lane 3–6: clinical isolates

The *icaA* and *icaD* genes were detected in 88.6% **(39/44)** of MRSA isolates and in 40.9% **(9/22)** of the MSSA isolates Figure 1.

The relation between the presence of *icaA* and *icaD* genes and phenotypic characterization of both MRSA and MSSA are presented in Table (4).

**Table 4:** Relation of phenotypic characterisation by CRA and MTP and genotypic identification of *IcaA* and *IcaD* genes

MRSA isolates				
CRA	MTP	*icaA* gene identification	*icaD* gene identification	MRSA isolates
Black	Strong Adherence	Positive	Positive	15
Black	Moderate Adherence	Positive	Positive	23
Red	Weak Adherence	Positive	Positive	1
Red	Negative	Negative	Negative	3
Black	Moderate adherence	Negative	Negative	2
			Total	44
			Total positive *ica* genes (No, %)*	39, 88.6%
**MSSA isolates**				
**CRA**	**MTP**	**icaA gene identification**	**icaD gene identification**	**MSSA isolates**
Black	Strong adherence	Positive	Positive	3
Black	Moderate Adherence	Positive	Positive	5
Red	Moderate adherence	negative	Negative	5
Red	Negative	Negative	Negative	8
Red	Weak adherence	Positive	Positive	1
			Total	22
			Total positive *ica* genes (No, %)*	9, 40.9%

*(Number,* percentage)*

CRA sensitivity was 95.8%, specificity 88.9%, 2 false negative and 2 false positive results were detected with Positive predictive value (PPV) 95.8%, negative predictive value (NPV) 88.9% and accuracy 93.9% compared to *icaA* and *icaD* detected by PCR Table 5.

**Table 5:** Comparative evaluation of qualitative CRA test for *S. aureus* strains biofilm detection in relation to *IcaA* and *IcaD* gene expression by PCR

CRA	PCR (*icaA* and *icaD*)		
	Positive	Negative	Total
Positive	46 (TP)	2 (FP)	48
Negative	2 (FN)	16 (TN)	18

*P*-value (*p* < 0.001)

MTP sensitivity was 100%, Specificity 61%, 7 false positive results were detected with a PPV of 87.3%, NPV of 100% and test accuracy 89.4% compared to *icaA* and *icaD* detected by PCR Table 6.

**Table 6:** Comparative evaluation of quantitative MTP test for *S. aureus* strains biofilm detection in relation to *IcaA* and *IcaD* gene expression by PCR

MTP	PCR (*icaA* and *icaD*)	
55 Positive	48 (TP)	7 (FP)
11 Negative	0 (FN)	11(TN)
Total:66		

*P*-value (*p* < 0.001)

## Discussion

MRSA remains a major public health concern globally, particularly due to its resistance to multiple antibiotics and its ability to spread in both healthcare and community environments. Early detection of MRSA carriers and timely implementation of infection prevention measures are essential steps to interrupt transmission and reduce the risk of infections.

As described by Raineri et al. in *“Staphylococcal trafficking and infection — from ‘nose to gut’ and back”*, the nasopharynx, oral cavity, and gut act as connected reservoirs for *S. aureus*, enabling movement and reseeding between sites. In mixed biofilm environments, these niches promote horizontal gene transfer and facilitate exchange of mobile *SCCmec* elements among S*taphylococci*, making transfer of *mec* determinants from nasal/oral strains into the broader human microbiome a reasonable concern [30].

Oral MRSA may therefore act as a local reservoir of methicillin-resistance determinants (*mec* genes) with potential to spread to other *Staphylococci* and, under certain conditions, to the wider microbiome. Mobile *SCCmec* elements carrying *mecA*/*mecC* are well documented to move between *Staphylococcal* species, and biofilm environments further promote such transfer through mechanisms including natural transformation and phage-mediated transduction [31].

Biofilms in the oral cavity thus create favorable conditions for gene exchange, and oral carriage of MRSA/*S. aureus* has been reported. Moreover, oral bacteria, including biofilm aggregates, are frequently swallowed and can reach the gastrointestinal tract, where they may transiently colonize or interact with gut bacteria. The gut microbiome, recognized as a major resistome, could therefore receive and maintain transferred *mec* determinants, extending the impact of nasal/oral reservoirs of resistance into the gastrointestinal tract [32].

This study evaluated the prevalence and characteristics of MRSA, among dental healthcare workers and patients across several dental clinics in Egypt, examining resistance profiles and biofilm-forming capabilities. The current data were collected from dental clinics within various dental settings, though the study does not aim to represent all clinical scenarios nationwide.

The study data revealed that 47.1% of the isolates were identified as *S. aureus*, with 66.6% of these isolates were identified as MRSA. MRSA Colonization rate of total isolates collected (44/200) was 22%, which is aligned with previous reports on nasal carriage in similar settings. For instance, Johnson et al. and Chmielowiec et al. reported colonization rates among healthcare workers ranging from 4% to 14% [33, 34]. Ukraine study revealed 31% *S. aureus* carriers among dental staff, with 31% of those being MRSA [35], while Lerche et al. observed 10.9% MRSA carriage among dental staff in Germany [36]. The previous data highlights the persistent risk of MRSA transmission in outpatient dental settings.

A global review of 22 studies (2014–2018) estimated an MRSA nasal carriage rate of 32.8% among healthcare workers [37], while a study from Saudi Arabia reported rates between 18% and 76% in different facilities [38]. These figures emphasize the urgent need for robust infection control measures, particularly in dental settings where regular MRSA screening (among DHCWs and patients attending dental clinics) and education may be lacking.

Regarding antibiotic resistance, none of the MRSA isolates were pan-drug resistant; however, they showed elevated resistance to several non-β-lactam antibiotics, notably clindamycin (72.7%) and erythromycin (52.3%). Resistance to gentamicin, ciprofloxacin, chloramphenicol, and tetracyclines was also observed. These results align with previous studies on MRSA colonizers [39], which found higher resistance in MRSA compared to MSSA. MRSA and MSSA isolates showed low resistance to vancomycin, amikacin, and linezolid, which is consistent with findings by Boncompain et al. [40]. Vancomycin resistance rate was low but should be interpreted with caution due to methodological limitations. Discrepancies in resistance rates, particularly to linezolid, may reflect population sampling differences, local antibiotic use patterns and microbiological detection method of resistance.

Biofilm formation was common among the isolates, with approximately 78% of *S. aureus* strains exhibiting moderate to strong biofilm formation. Among MRSA isolates, this rate increased to 95.4%, compared to 72.2% in MSSA. These findings are consistent with Omidi et al., who reported 93.1% biofilm production among *S. aureus* isolates from non-clinical samples [27]. Additionally, a study from Iran reported biofilm formation in 97.5% of MRSA strains and 60% in MSSA strains [41]. Lower biofilm rates (61%) have been noted in healthy adults [34], indicating that biofilm production is more common in healthcare-associated environments.

PCR confirmed that all MRSA isolates carried the *mecA* gene. Additionally, 88.6% of MRSA isolates were positive for both the *icaA* and *icaD* genes, associated with biofilm formation via PIA. These results agree with previous studies, such as Salmanov et al., who found universal *mecA* positivity in MRSA-colonized healthcare workers in Ukraine [35]. Higher *ica* gene carriage has also been reported by Ohadian Moghadam et al. in inpatient-derived colonizers [41].

To validate biofilm phenotyping, both CRA and microtiter plate assays were compared to PCR results. CRA exhibited 95.8% sensitivity and 88.9% specificity, while MTP demonstrated 100% sensitivity but lower specificity at 61%. These results are consistent with previous studies [42]. However, Omidi et al., noted that a small proportion of MRSA isolates lacking *ica* genes still produced biofilm [27], suggesting that other mechanisms may contribute to this virulence factor.

## Conclusions

The high MRSA colonization rates among DHCWs and patients, along with significant antibiotic resistance and biofilm-forming abilities, highlight the urgent need for regular screening and enhanced infection control measures in dental healthcare settings. Future research should aim to expand sampling and clarify more site selection criteria to better inform infection control strategies and improve generalizability.

### Study limitations

One of the main limitations of our study was related to sample collection, from dental clinics across various regions in Egypt. The variability between clinics and geographic locations posed logistical challenges. Future studies should expand on sample size and incorporate environmental surveillance to better delineate the transmission dynamics within outpatient dental settings. Additionally, the high patient-to-dental staff ratio limited our ability to obtain a more balanced representation. Another limitation is that, since the study was primarily focused on MRSA isolates, we did not perform vancomycin MIC testing to determine the exact susceptibility or resistance patterns. This limitation could be addressed in future research exploring vancomycin resistant *S. aureus* isolates from dental settings in Egypt.

## Electronic Supplementary Material

Below is the link to the electronic supplementary material.

Appendix 1 (The medical questionnaire)

## Data Availability

The data that support the findings of this study are available from the corresponding author, [R.A], upon reasonable request.

## Abbreviations

CRA: Congo red agar
DHCWs: Dental healthcare workers
MRSA: Methicillin-Resistant *Staphylococcus aureus*
MSSA: Methicillin-Sensitive *Staphylococcus aureus*
MTP: Microtiter plate method
OD: Optical density
ODc: Cut-off optical density
PCR: Polymerase Chain Reaction
PIA: Polysaccharide intercellular adhesin protein
S. aureus: *Staphylococcus aureus*
