# Supplementary material for: Detection of Methicillin-resistant Staphylococcus aureus (MRSA) and biofilm formation among dental patients and dental health care workers: cross sectional study
Source: Clin Oral Investig. 2026 Jan 3;30(1):36. doi: 10.1007/s00784-025-06684-9 (PMC12764656; doi:10.1007/s00784-025-06684-9)
Supplement: Supplementary file 1 — Appendix 1 (The medical questionnaire) [file 784_2025_6684_MOESM1_ESM.docx]

**MEDICAL QUESTIONNAIRE**

**Please read carefully and answer most accurately.**

**Personal data of the participant**

First and last name:

Date of birth:

Address:

City, country:

Phone:

Profession:

Passport or ID number:

**Medical information**

Body weight (kg): Height (cm):

Are you a smoker? □ YES □ NO

If your answer is YES, how much are you smoking on a daily basis?

Are you drinking alcohol? □ OFTEN □ OCCASIONALLY □ NO

Have you previously been hospitalized? □ YES □ NO

Have you ever taken (mupirocin - mupirocin saja - bactroban - centany - bactroform - probactin - mupirax - teriaswab) nasal cream? □ YES □ NO

If your answer is YES, how long did you use it?........................................................

Are you taking any medications on a regular or occasional basis? □ YES □ NO

If your answer is YES; please write all the medications, you take:

| Medication name | Dosage | How often on a daily basis |
| --- | --- | --- |
|  |  |  |
|  |  |  |
|  |  |  |
|  |  |  |

Did you take any antibiotics before? □ YES □ NO

If your answer is YES; please write the antibiotics you used

| Medication name | Dose frequency  (Once, twice daily…etc.) | Duration | Was it prescribed by a physician or self-prescribed |
| --- | --- | --- | --- |
|  |  |  |  |
|  |  |  |  |
|  |  |  |  |
|  |  |  |  |

Are you taking any herbal and natural medications? (Ginseng, garlic and similar) □ YES □ NO

If your answer is YES, please write what medications?

Have your body temperature raised, cold or flu in last month? □ YES □ NO

Have you been severely diseased in the last 2 years? □ YES □ NO

If your answer is YES, please write about it:

Please write about your previous surgeries (if you had them), and when you were operated on:

**For participants working in the medical field**

How many patients do you come in contact with on a daily basis? And what are your regular precautions to prevent infections?

I, signed below, state that I fully understood all the questions, received needed explanations and that data in this questionnaire is authentic.

Date: Signature:
